# Supplementary material for: Impact of Pain Education on Pain Relief in Oncological Patients: A Narrative Review of Systematic Reviews and Meta-Analyses
Source: Cancers (Basel). 2025 May 16;17(10):1683. doi: 10.3390/cancers17101683 (PMC12110527; doi:10.3390/cancers17101683)
Supplement: Supplementary file 1 [file cancers-17-01683-s001.zip › cancers-3582373-supplementary.pdf]

Supplementary table S1: narrative review checklist

Section/topic	#	Checklist item	Reported on page or line #
**TITLE**			
title	1	Identify the report as a Narrative Review	Page 1
**ABSTRACT**			
Unstructured summary	2	Provide an unstructured summary including, as applicable: background. Objective, brief summary of narrative review and implications for future research, and clinical practice or policy development.	Page 2
**INTRODUCTION**			
Rationale/background	3	Describe the rationale for the review in the context of what is already known	Page 3
Objectives	4	Specify the key question(s) for the review topic	Page 3
**METHODS**			
Research selection	5	Specify the process for identifying the literature search (e.g. years considered, language, publication status, study design, and databases of coverage	Page 3
**DISCUSSION/SUMMARY**			
Narrative	6	Discuss: 1) research reviewed including fundamental or key findings, 2) limitations and/or quality of research reviewed, and 3) need for future research.	Page 6
Summary	7	Provide and overall interpretation of the narrative review in the context of clinical practice for health professionals, policy development and implementation, or future research.	Page 7

Supplementary table S2: Summary of quality evaluation of the included reviews based on AMSTAR 2 domains

AMSTAR-2 domain	Authors, publication year [reference]								
	Allard *et al.* 2001 [8]	Goldberg *et al.* 2007 [9]	Bennett *et al.* 2009 [14]	Oldenmenger *et al.* 2009 [10]	Cummings *et al.* 2011 [15]	Ling *et al.* 2011 [11]	Koller *et al.* 2012 [12]	Jho *et al.* 2013 [16]	Oldenmenger *et al.* 2018 [13]
Review question (PICO)	N	N	Y	N	Y	Y	Y	Y	N
A priori protocol*	N	N	N	N	Y	N	N	N	N
Study designs included/explained	Y	Y	Y	Y	Y	Y	Y	Y	Y
Comprehensive literature search*	Y	Y	Y	Y	Y	Y	Y	Y	Y
Duplicate study selection	Y	Y	Y	Y	Y	Y	N	Y	Y
Duplicate data extraction	Y	Y	Y	Y	Y	Y	N	Y	Y
List and justification of excluded studies*	N	N	N	Y	Y	Y	Y	Y	Y
Adequate description of included studies	Y	Y	Y	Y	Y	Y	Y	Y	Y
Satisfactory technique for assessing RoB in non-RCTs*	Y	Y	Y	N	Y	Y	Y	N	Y
Funding sources reported for included studies	N	N	N	N	N	N	N	N	N
Appropriate methods for quantitative synthesis*	NA	NA	Y	NA	Y	NA	NA	Y	NA
Impact of RoB on pooled results	NA	NA	P	NA	Y	NA	NA	Y	NA
Impact of RoB on interpretation of pooled results*	N	N	P	N	Y	N	N	N	NA
Heterogeneity explained	N	P	Y	N	Y	P	P	Y	Y
Publication (small study) bias assessed*	NA	NA	N	N	Y	NA	NA	Y	Y
Funding sources and conflict of authors reported	Y	Y	Y	Y	Y	Y	Y	Y	Y
**Overall confidence rating**	**CL**	**CL**	**CL**	**CL**	**H**	**CL**	**CL**	**CL**	**L**

Legend: *: critical domains in AMSTAR 2; CL: critically low; H: high; L: low; N: no; NA: not applicable because a meta-analysis was no conducted; P: partial yes; PICO: population, indication, comparator, outcomes; RCTs: randomized controlled trials; RoB: risk of bias; Y: yes.
